# Enhancing Psychiatric Care for Older Adults in a Care Home Setting: A Quality Improvement Project

**DOI:** 10.1192/bjo.2025.10399

**Published:** 2025-06-20

**Authors:** Alex Milone, Yasir Hameed, Trish Phiri, Peace Ayodele

**Affiliations:** NSFT, Norwich, United Kingdom

## Abstract

**Aims:** The aims of the project were (i) to enhance collaboration between the multidisciplinary team working with people living with dementia such as the carers, general practitioners, pharmacists, mental health professionals and patients` families; (ii) improve the Community Mental Health Team (CMHT) response to referrals; (iii) reduce unnecessary referrals; (iv) provide psychiatric training to primary care colleagues; (v) reduce care home visits; (vi) reduce polypharmacy, especially antipsychotics in dementia; and (vii) increase the use of memory medications for dementia patients where indicated.

**Methods:** A pilot project was conducted with a care home specialising in dementia with a high rate of referrals to secondary mental health services. A fortnightly MDT meeting was set up which included care home staff, CMHT staff, GP staff and family members. Data was then captured from notes for analysis, with corroboration from the electronic patient record (EPR) as necessary.

**Results:** The project involved 64 patients with various psychiatric and dementia-related needs. The median age of the patients was 86, with a male to female ratio of 36:64. The project addressed a range of symptoms of concern, with 95% of patients exhibiting Behavioural and Psychological Symptoms of Dementia (BPSD), and the remaining 5% presenting with other issues such as seizures, elevated prolactin levels, and memory decline. Over the course of the project, there were a total of 317 patient discussions.

There was a 25% reduction in referrals to the CMHT in the 24-month period after MDT was started. Medication management was a significant focus, with 11 patients starting melatonin, 45 patients starting other medications, 22 patients starting antipsychotics, and 12 antipsychotic reviews conducted. Additionally, 10 patients had their antipsychotics stopped, 29 memantine titrations were performed, and 5 acetylcholinesterase inhibitors (AChEi) titrations were completed. Physical health monitoring, including prolactin and ECG checks, was conducted for 28 patients.

Family involvement was a key component, with 21 out of 64 patients having family members participate in the MDT meetings. Palliative care discussions were held for 5 patients, and there were 6 referrals to the Intensive Older People’s Service (IOPS) and 2 referrals to the Memory Treatment Service.

**Conclusion:** This pilot study demonstrated the effectiveness of a regular MDT for a care home with a high referral rate to secondary care, by reducing the number of referrals, improving communication between services and optimising the medical treatment of BPSD. This model shows promise for broader implementation to enhance the quality of psychiatric care for older adults in care home settings.

